# ‘A Meaningful Difference, but Not Ultimately the Difference I Would Want’: A Mixed‐Methods Approach to Explore and Benchmark Clinically Meaningful Changes in Aphasia Recovery

**DOI:** 10.1111/hex.14169

**Published:** 2024-08-06

**Authors:** Sally Zingelman, Dominique A. Cadilhac, Joosup Kim, Marissa Stone, Sam Harvey, Carolyn Unsworth, Robyn O'Halloran, Deborah Hersh, Kathryn Mainstone, Sarah J. Wallace

**Affiliations:** ^1^ School of Health and Rehabilitation Sciences, Queensland Aphasia Research Centre The University of Queensland St Lucia Queensland Australia; ^2^ Surgical Treatment and Rehabilitation Service (STARS) Education and Research Alliance The University of Queensland and Metro North Health Herston Queensland Australia; ^3^ Centre of Research Excellence in Aphasia Recovery and Rehabilitation La Trobe University Melbourne Victoria Australia; ^4^ Stroke and Ageing Research, Department of Medicine School of Clinical Sciences at Monash Health, Monash University Clayton Victoria Australia; ^5^ Stroke Division The Florey Institute of Neuroscience and Mental Health Heidelberg Victoria Australia; ^6^ St Vincent's Hospital Melbourne Fitzroy Victoria Australia; ^7^ Department of Medicine School of Clinical Sciences at Monash Health, Monash University Clayton Victoria Australia; ^8^ Institute of Health and Wellbeing Federation University Ballarat Victoria Australia; ^9^ Discipline of Speech Pathology, School of Allied Health, Human Services and Sport La Trobe University Bundoora Victoria Australia; ^10^ Speech Pathology, Curtin School of Allied Health Curtin University Perth Western Australia Australia; ^11^ School of Allied Health Science and Practice University of Adelaide Adelaide South Australia Australia

**Keywords:** aphasia, interpretation, meaningful change, minimal important change, outcome measures

## Abstract

**Introduction:**

Outcome measurement instruments (OMIs) are used to gauge the effects of treatment. In post‐stroke aphasia rehabilitation, benchmarks for meaningful change are needed to support the interpretation of patient outcomes. This study is part of a research programme to establish minimal important change (MIC) values (the smallest change above which patients perceive themselves as importantly changed) for core OMIs. As a first step in this process, the views of people with aphasia and clinicians were explored, and consensus was sought on a threshold for clinically meaningful change.

**Methods:**

Sequential mixed‐methods design was employed. Participants included people with post‐stroke aphasia and speech pathologists. People with aphasia were purposively sampled based on time post‐stroke, age and gender, whereas speech pathologists were sampled according to their work setting (hospital or community). Each participant attended a focus group followed by a consensus workshop with a survey component. Within the focus groups, experiences and methods for measuring meaningful change during aphasia recovery were explored. Qualitative data were transcribed and analysed using reflexive thematic analysis. In the consensus workshop, participants voted on thresholds for meaningful change in core outcome constructs of language, communication, emotional well‐being and quality of life, using a six‐point rating scale (much worse, slightly worse, no change, slightly improved, much improved and completely recovered). Consensus was defined a priori as 70% agreement. Voting results were reported using descriptive statistics.

**Results:**

Five people with aphasia (*n* = 4, > 6 months after stroke; *n* = 5, < 65 years; *n* = 3, males) and eight speech pathologists (*n* = 4, hospital setting; *n* = 4, community setting) participated in one of four focus groups (duration: 92–112 min). Four themes were identified describing meaningful change as follows: (1) different for every single person; (2) small continuous improvements; (3) measured by progress towards personally relevant goals; and (4) influenced by personal factors. ‘Slightly improved’ was agreed as the threshold of MIC on the anchor‐rating scale (75%–92%) within 6 months of stroke, whereas after 6 months there was a trend towards supporting ‘much improved’ (36%–66%).

**Conclusion:**

Our mixed‐methods research with people with aphasia and speech pathologists provides novel evidence to inform the definition of MIC in aphasia rehabilitation. Future research will aim to establish MIC values for core OMIs.

**Patient or Public Contribution:**

This work is the result of engagement between people with lived experience of post‐stroke aphasia, including people with aphasia, family members, clinicians and researchers. Engagement across the research cycle was sought to ensure that the research tasks were acceptable and easily understood by participants and that the outcomes of the study were relevant to the aphasia community. This engagement included the co‐development of a plain English summary of the results. Advisors were remunerated in accordance with Health Consumers Queensland guidelines. Interview guides for clinicians were piloted by speech pathologists working in aphasia rehabilitation.

## 1 Introduction

Aphasia is an acquired language disorder that impacts communication. It is estimated that as many as 40 million people globally live with post‐stroke aphasia [1, 2, 3]. Aphasia affects people differently, with changes experienced across speaking, reading, writing and understanding others. The impacts of aphasia on language, communication (i.e., the use of language to participate in daily activities), emotional well‐being and quality of life are significant [4]. Indeed, these were the basis for the development of the Research Outcome Measurement in Aphasia Core Outcome Set (ROMA COS) [5, 6, 7, 8].

Although a COS for aphasia has been agreed upon, there is a lack of guidance about how to best interpret meaningful change using outcome measurement instruments (OMIs) [9, 10]. One approach to defining clinically meaningful changes—that is, the difference in how an individual functions or feels—is minimal important change (MIC) [11, 12, 13, 14]. MIC is defined as the smallest numeric difference in an OMI that is considered clinically meaningful by patients [11, 12]. MIC values are established using an anchor‐based analysis that pairs OMI scores together with a patient‐rated external criterion or anchor. To interpret the amount of change that is clinically meaningful, anchor‐based MIC studies require patient perceptions of change on the anchor‐rating scale and a threshold on this scale that defines *minimal importance* for the population of interest [15, 16]. An example of a mixed‐methods approach to establishing both of these factors comes from Dubbelman et al. [17]. They conducted a qualitative survey with clinicians and caregivers of people with Alzheimer's disease and related disorders to define thresholds for decline and improvement. Subsequently, they used statistical analyses to establish MIC values for the OMI of interest, the Amsterdam Instrumental Activities of Daily Living Questionnaire [17]. Using this mixed‐methods approach, they produced a MIC value based on stakeholder perspectives of meaningful change.

The Collaboration of Aphasia Trialists (https://www.aphasiatrials.org/) has identified developing MIC values for the ROMA COS OMIs as an international priority [18]. However, to date, only one value of clinically meaningful change has been established for the Singapore version of the Stroke and Aphasia Quality of Life Scale‐39g. Neither patient perceptions of change nor patient‐defined thresholds informed this process. Consequently, there are currently no benchmarks that represent clinically meaningful change from the perspectives of people with aphasia [19]. The objectives of this study were as follows: (1) to explore the perspectives of people with aphasia and speech pathologists about clinically meaningful changes in aphasia recovery, and (2) to establish stakeholder consensus on thresholds for meaningful change using anchor‐rating scales for core aphasia OMIs. This is the first step in establishing MIC values for the ROMA COS.

## 2 Materials and Methods

### 2.1 Study Design

**Figure 1:**
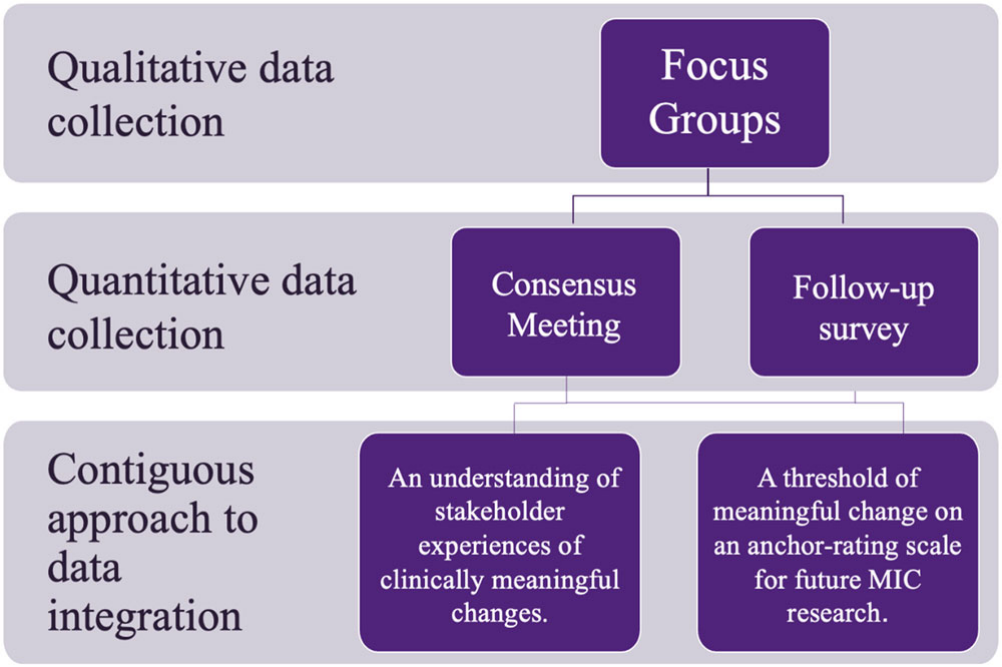
Sequential mixed‐methods study design.

A sequential mixed‐methods study was conducted in two stages (Figure 1). First, a series of online focus groups were conducted to explore stakeholder perspectives of clinically meaningful changes in aphasia recovery. Second, quantitative data were collected via a voting process in an online workshop and a follow‐up survey. Data collection was conducted using Zoom videoconferencing version 5.16.10 [20] and Qualtrics web‐based survey software [21] in April and May 2023. This study is reported in line with the People with Aphasia and Other Layperson Involvement (PAOLI) framework (File S1) [22].

#### 2.1.1 Ethics and Data Availability

Ethical approval was obtained from the University of Queensland's Human Research Ethics Committee (2022HE001946). Data that support the findings of this study are not publicly available. Anonymised data may be made available from the corresponding author upon reasonable request.

### 2.2 Participants

#### 2.2.1 Sampling and Recruitment

Participants included people with post‐stroke aphasia and speech pathologists working in aphasia rehabilitation. Purposive sampling was used to recruit participants with aphasia with variation in time after stroke (1 week to 6 months; ≥ 6 months), age (< 65 years; ≥ 65 years) and gender (male; female). The characteristics of participants with aphasia are described in line with established reporting standards in Table 2 [23]. Speech pathologists were sampled to achieve variation in workplace (hospital or community settings). Individuals with aphasia from an aetiology other than stroke, or who had conditions that impacted their ability to participate in the focus groups (e.g., dementia, severe vision or hearing impairment, severe neurological condition or inability to verbally or non‐verbally indicate a yes/no response) were excluded from the study.

Participants were recruited Australia‐wide through stroke and aphasia professional organisations, including the Australian Aphasia Association, Queensland Aphasia Research Centre, Stroke Foundation, the Centre of Research Excellence in Aphasia Recovery and Rehabilitation and the Speech Pathology Email ChatS (SPECS) Google group. Written project information was presented using accessible language and formatting for people living with aphasia [24]. Interested individuals were invited to submit an expression of interest via Qualtrics. Prospective participants with aphasia then attended an individual videoconferencing meeting. In this meeting, the participant information and consent were discussed verbally with pictorial supports, with opportunities to ask questions before obtaining informed consent. Prospective speech pathologist participants were provided with written consent forms via email.

### 2.3 Procedures

#### 2.3.1 Anchor‐Rating Scale Development

**Figure 2:**
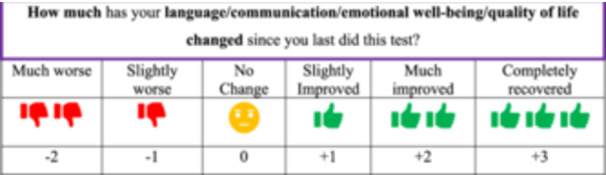
Anchor‐rating scale.

An anchor‐rating scale was developed by the research team (Figure 2). To establish MIC values, anchor‐rating scales are completed at the post‐treatment time point alongside OMIs (or ‘tests’). The development of an anchor required consideration of two components: first, an anchor question that was conceptually relevant to the constructs of interest, easily understood by patients and inclusive of a specific time frame [10, 12, 25, 26]; and second, a scale for measuring patient‐rated responses. Breitenstein et al. proposed the use of novel anchor questions for evaluating aphasia treatments, such as ‘How much has your [construct] changed since your last visit/treatment started’, which formed the foundation for our anchor‐rating scale [10, p. 1718]. Additionally, it was suggested to measure patient responses using the six‐point Likert scale, as established by Revicki and colleagues [10, 26]. The proposed Likert scale was reviewed [26]. An indicator for completely recovered (+3) was considered important to include, as a full recovery from aphasia is a possible outcome for some patients after stroke [27]. However, an acceptable and clinically feasible indicator for a point lower than much worse (−3) could not be identified. As such, the final rating scale was developed with six points to represent changes in aphasia recovery and a question time frame relevant to the planned pre–post‐treatment study (‘since you last did this test’). The overall question wording was refined in response to consumer piloting (File S2).

#### 2.3.2 Focus Groups

Four focus groups were held to allow participants with shared experiences (e.g., time after stroke or work environment) to interact and discuss their experiences about clinically meaningful changes in aphasia recovery (Table 1). Group sizes were small to allow participants adequate time to understand and discuss the abstract concepts related to meaningful change and the importance of changes experienced. Each group was facilitated by the first researcher (S.Z.) and supported by the lead researcher (S.J.W.). Focus groups were recorded using Zoom's built‐in recording function.

**Table 1:** Summary of participants and procedures.

Data collection method	Participant groups	Topic guide summary	Duration
Focus Group 1	People with aphasia 5–8 months after stroke	1. Importance of changes experienced. 2. Amount of change experienced. 3. Amount of change that made a meaningful difference.	105 min
Focus Group 2	People with aphasia more than 8 months after stroke		110 min
Focus Group 3	Speech pathologists working in community settings	1. The concept of clinically meaningful change. 2. Current outcome measurement practices. 3. Amount of change that made a meaningful difference.	111 min
Focus Group 4	Speech pathologists working in hospital settings		91 min
Consensus workshop and survey	All people with aphasia and speech pathologists	1. Overall, what is the smallest amount of change that is important? 2. Is important change different at different stages (< 6 months after stroke, > 6 months after stroke) of aphasia recovery? 3. Is important change different for different severities of aphasia?	86 min

A topic guide (as summarised in Table 1) provided a framework for the discussion. The topic guide was developed from the literature and then refined by pilot testing with an advisor with aphasia and two speech pathologists. In the focus groups, information was shared to explore important concepts, including clinically meaningful change, language, communication, emotional well‐being and quality of life. The relevance of the anchor‐rating scale was explained. Supported communication strategies were used, including multimodal communication, written keywords, additional processing time for comprehension and response generation, use of fixed‐choice questions and verification of responses [28]. Additionally, participants could elect to bring a friend or family member to the focus groups for additional support.

#### 2.3.3 Consensus Workshop

Following the focus groups, participants were invited to attend a single consensus workshop (Table 1). Before the workshop, key concepts and topics of discussion from the focus groups were summarised in an aphasia‐friendly video and disseminated to all participants. In the workshop, the focus group discussions were summarised, followed by testing of Zoom's built‐in poll for accessibility for all participants. The consensus workshop was facilitated by the first researcher (S.Z.) and supported by other members of the research team (S.J.W., C.U. and K.M.). Quantitative data were recorded in a Zoom polling report.

Participants voted anonymously using the Zoom polling function to answer key questions required to establish thresholds on the anchor‐rating scales. A total of 20 questions were developed by the research team (Table 1), which were piloted by an advisor with aphasia and two speech pathologists. Nine questions about language and communication were presented in the consensus meeting. Results were reviewed after each poll. An iterative process was followed to seek consensus. Where consensus was not reached, open discussion between the participants was invited, and the question and voting process were repeated. One question asking about aphasia severity was presented in the workshop; however, participants reported the question was unclear. Subsequently, the four planned questions relating to aphasia severity were removed from the agenda and excluded from the voting process.

Not all planned questions were able to be completed in the time allocated for the consensus workshop. Participants decided that there had been sufficient discussion generated within the workshop to respond to the remaining questions independently via an online follow‐up survey. The eight remaining questions, which were focused on different constructs, were the same questions presented in the consensus meeting. Communication support was offered to participants with aphasia whereby individual meetings with the first author were scheduled over Zoom. Verbal and pictorial support were provided to support participants' understanding of the survey questions. Participants selected their responses on a Qualtrics survey on their own devices; therefore, voting anonymity was maintained. Speech pathologist participants were emailed the same Qualtrics survey for completion.

### 2.4 Data Analysis

#### 2.4.1 Qualitative Analysis

To understand experiences of clinically meaningful changes, a qualitative descriptive approach guided the analysis, conducted by author S.Z. [29]. Focus group data were analysed using the six‐phase reflexive thematic analysis outlined by Braun and Clarke [30]:

1. *Familiarisation*: Recordings were transcribed verbatim using UQ CalPy Online v2.1 [31] for transcription. Transcriptions were reviewed against the recordings to ensure accuracy and were corrected as required. This process enabled repeated immersion in the data. Observations of emerging patterns were documented.
2. *Data coding*: Data were initially coded as four separate data sets, one for each focus group. Transcripts were read in full. Data units that conveyed meaning towards the research questions were colour‐coded and given a short, descriptive label. Codes were iteratively revised during this phase.
3. *Generating initial themes*: Initial themes were identified based on the patterns identified in the codes. The codes were arranged together and summarised into tentative themes to further explore the identified patterns in the data.
4. *Reviewing and developing themes*: The categories were revised and relationships between codes and categories were explored in a visual mind map. During this process, strong patterns were observed between codes generated within each of the two participant groups. Recordings and transcriptions were reviewed, and two data sets were formed: one with transcriptions from people with aphasia and one from speech pathologists. Whether all four transcripts could be collated and analysed together was explored. However, as there was a difference in the questions asked of each participant group (Table 1), the resulting data captured distinct perspectives relating to clinically meaningful changes. The two data sets were analysed separately, resulting in different categories and themes from each. The themes and categories were revised for consistency in meaning and coding.
5. *Refining, defining and naming themes*: Consistency of scope, labelling, and meaning within each of the themes were revised in relation to the research questions for relevance by authors S.Z. and K.M., together with members of the consumer advisory group (further details in File S1). Together, research team members brought their personal positioning (below) to the analysis. In doing so, we sought to describe the depth of the participants' experiences, rather than present them as a singular truth, consistent with a reflexive approach.
6. *Writing the report*: The final themes, categories and sub‐categories were described with examples and anonymised with the use of participant codes.

#### 2.4.2 Researcher Characteristics and Reflexivity

The research team comprised researchers with experience using qualitative research methods and working clinically with people with aphasia (S.Z., S.J.W., M.S., S.H., C.U., R.O'H. and D.H.), in stroke and health services research (D.A.C. and J.K.), and with lived experience of aphasia (K.M.).

Data collection and analysis were led by the first author (S.Z.). S.Z. is a speech pathologist with prior experience in qualitative research. She had previous interactions with one participant through a community aphasia group. Clinically, S.Z. adopts a functional approach to working with people with communication disorders, which informed her viewpoint what is considered meaningful may be unique to the circumstances and goals of each person.

#### 2.4.3 Quantitative Analysis

In the consensus process, questions were asked with either yes/no responses or multiple choices from the six indicators on the anchor‐rating scale (Figure 1). Votes were reported using descriptive statistics (percentages of responses). Consensus was defined as ≥ 70% agreement, in keeping with previous consensus studies in aphasia research [7, 9, 23, 32, 33]. A contiguous approach to data integration was taken, whereby both the qualitative and quantitative results are presented within this single study, in separate sections [34].

## 3 Results

Of the 20 participants who consented to participate in the study, five people with aphasia and eight speech pathologists participated in a focus group. One speech pathologist was unavailable for the second stage of the study, resulting in a total of 12 participants voting in the consensus process. Characteristics of participants with aphasia are detailed in Table 2. Speech pathologist participants were female (100%) and reported 2–30 years of experience working in aphasia rehabilitation across acute, subacute and community settings. One family member attended Focus Group 2 and the consensus workshop as additional communication support.

**Table 2:** Characteristics of participants with aphasia.

Participants with aphasia	PWA01	PWA02	PWA03	PWA04	PWA05
Gender	Female	Male	Male	Male	Female
Age (years)	47	56	56	61	47
Location	Queensland	Victoria	New South Wales	Queensland	Victoria
Primary language	English	English	English	English	English
Additional language/s	No	No	No	No	No
Full‐time education (years)	17	a	11	15	16
History of conditions impacting communication/cognition	No	No	No	No	No
Stroke					
Lesion hemisphere	Left	Left	Left	Left	Left
Time since aphasia onset (years; months)	0; 5	0; 8	4; 1	1; 7	16; 11
Previous stroke	No	No	No	No	No
Conditions arising after stroke					
Apraxia of speech	Yes	Yes	Yes	Yes	Yes
Dysarthria			Yes		
Hemiplegia				Yes	
Hemiparesis	Yes		Yes	Yes	Yes
Cognition changes	Yes	Yes		Yes	Yes
Depression				Yes	
Fatigue				Yes	Yes

^a^ Missing data.

Four themes were identified. Meaningful changes were described by people with aphasia as (1) different for every single person and (2) small continuous improvements. Congruently, speech pathologists defined meaningful changes as (3) measured by progress towards meaningful goals and (4) influenced by personal factors (Co‐developed, aphasia‐friendly summary in File S2) (Table 3).

**Table 3:** Summary of themes, categories and supporting quotes.

Themes	Categories	Sub‐categories	Example supporting quotes
Meaningful changes in aphasia recovery are:			
Different for every single person	Individual experiences of recovery	Language	*For the first 6 months I couldn't talk, but I learnt people's names. Now my talking is ok.* (PWA01)
		Communication	*Couple of months ago I didn't answer the phone. But it is mighty important to answer the phone*. (PWA04)
	A new normal	Quality of life Emotional well‐being	*My wife says, well, we can't go out like we used to do. So we'll do different things, you know? And so I thought, oh, that's cool. That's cool. I like that. I'm happy with that.* (PWA02)
	Mindset	Quality of life Emotional well‐being	*I can't think about what I've lost because I've got another half a life to live, so gotta find some other meaningful way*. (PWA05)
Small continuous improvements	Motivating	Important to celebrate	*I call my wife some days and say ‘you should see this, I did this today’ … It's a happy day*. (PWA02)
	The result of persistence	Experiences of rehabilitation Patience Frustration	*Some days you get you sort of go, I'm done with it. I'm you know, I just had enough of it, but you just gotta get past it. That was a really big thing from the standing, walking, being able to speak, that's a big thing.* (PWA02)
	Every step is important	Short‐term goals Small improvements	*It's immensely important to step from one improvement to the next.* (PWA04)
Progress towards personal goals	Measuring the patient perspective	Patient‐reported outcome measures Standardised assessments Informal rating scales	*I have a strong preference for the patient perspective … because I think that's what's really important.* (SP06)
	Measuring functional and personal relevance	Clinical observations Observations from significant others	*I'll notice [patient] needs less prompting, the time it takes, measuring the more functional change in patient directed areas and tasks*. (SP08)
	Collaborative goal setting	Person‐centred care	*It starts with the goal setting process, making sure the patient's involved. Helping them to see and identify their progress towards goals. Making the goals tangible and meaningful enough to measure change*. (SP06)
Influenced by personal factors	Individual factors	Age Time since stroke Expectations Insight Impact of a change	*Experiences of change are so individual … depends on coping skills, relationships, ability to adjust.* (SP06)
	Social determinants of health	Languages spoken Cultural expectations Health literacy	*There are cultural influences on what people see as meaningful.* (SP06)
	Impact of a change	Participation Usual activities	*Severity and impairment does not equal impact. Impact is more fundamental [to meaningful change] than anything else.* (SP03)

### 3.1 Theme 1: Meaningful Changes Are Different for Every Single Person

Participants with aphasia recalled experiences that illustrated meaningful changes throughout their recovery. However, it was emphasised that these experiences were important to participants personally and may not be meaningful to others: ‘I think that the challenge [is] it [aphasia] is different for everybody I know’ (PWA04). The individual nature of change was highlighted in the diversity of meaningful experiences that participants recalled across the concepts of language, communication, quality of life and emotional well‐being. Participant PWA03 described the meaningful change experienced across their language and communication in the 4 years since their stroke: ‘I couldn't say anything, I couldn't, only noises. That's all I could say. Just noises … it's important to speak again … it was much improved and I still have a little way to go.’

Adjustment to post‐stroke life was framed as a positive improvement in quality of life, described as ‘It's not going to be the same as before stroke with what I was doing, has to be a new normal’ (PWA01). Examples of new, but meaningful, changes after stroke included recreational activities: ‘I am more active now since my stroke’ (PWA03); ‘I started to really enjoy painting’ (PWA01); and ‘photography which you know, I was always gonna do that next week. Well now I can do it. I'm having fun’ (PWA04). Connection in support groups was described as part of ‘the new normal’ after the stroke leading to meaningful improvements: ‘The aphasia group is amazing … the support that they've given me and the friendship’ (PWA05).

Participants described how shifts in mindset led to positive improvements in emotional well‐being:

The biggest problem to someone like me, I've gone from I'm running around the boss of everything … and then all of a sudden you just wake up one morning and go ‘What? What am I doing now?’ And I can't stand. I can't speak and I can't wash myself. You know, you actually think I'd rather be dead … I don't know what it was, but there was a point where you gotta go … . Well, there's a lot of other reasons you should be here as well. So and then you sort of had to go from that [depression] to … getting bigger and better. And now I actually sort of feel now I get really excited about doing things I go, wow, that's a that's a good day!

(PWA04)

Speech pathologists also described the individual nature of change during recovery. However, the scope of their descriptions was more specific to the personal factors that may influence the perception of meaningful change and are detailed in theme 4.

### 3.2 Theme 2: Meaningful Changes Are Small, Continuous Improvements

Small, continuous improvements were emphasised as important by participants with aphasia, regardless of the type of change. Participants with aphasia spoke about the importance of celebrating small improvements. They elaborated that recognition of achieving small goals was motivating, particularly since engagement with rehabilitation activities required persistence and patience. However, participants also emphasised that small, yet important, improvements are not the ultimate desired outcome of aphasia treatments, with one participant stating improvements were: ‘a meaningful difference, but not ultimately the difference I would want’ (PWA02). Rather than quantifying the amount of change that might be meaningful, speech pathologists agreed that meaningful change ‘depends on the patient and what their goals are [for communication recovery]’ (SP01).

### 3.3 Theme 3: Meaningful Changes Are Measured by Progress Towards Personal Goals

Speech pathologist participants viewed measuring meaningful change through the lens of patient‐centred care. Collaborative goal setting and documenting progress towards individualised goals emerged as a central process for measuring meaningful changes. Participant SP07 explained: ‘So, getting them to rate their own individualised goals and seeing that improvement from their perspective is something that I would see as a clinically meaningful change’. Collecting baseline information was described as a starting point for understanding the individual patient, with variation in preferred OMIs reported, including standardised assessments, patient‐reported outcomes and informal rating scales. The collation of observations outside the treatment setting was considered valuable to understanding a person's independence in usual activities, with family members, support workers and multidisciplinary observations all reported as informing treatment. It was noted by the investigators facilitating the focus groups that goal setting was not raised in focus group conversations led by people with aphasia.

### 3.4 Theme 4: Perceptions of Meaningful Change May Be Influenced by Personal Factors

The impact of a change on a person with aphasia's day‐to‐day activities and participation was discussed by speech pathologists as a salient factor in whether a change was perceived as meaningful. By way of example, a retired person residing at home with a long‐term partner was described as ‘being able to do everything they did before’, whereas another person living independently and previously employed in a role with high communication demands may require a greater amount of language improvement before it is perceived as meaningful. Ultimately, speech pathologist participants emphasised that ‘Experiences of change are so individual’ (SP07). This sentiment was reiterated by one participant with aphasia, who stated: ‘Even though I understand what other people are going through because I've been there, done that, I don't really understand because there'd be other types of things in their lives that are different’ (PWA05).

### 3.5 Consensus‐Defined Thresholds of Minimal Importance

All participants agreed that the minimum amount of meaningful change varies by time after stroke. In the first 6 months after a stroke, consensus (≥ 70% agreement) was reached. ‘Slightly improved’ was the agreed indicator of meaningful change in aphasia recovery for the constructs of language, communication, emotional well‐being and quality of life on the six‐point anchor‐rating scale. Consensus was not reached on an indicator of meaningful change in any of the four constructs for changes occurring more than 6 months after stroke. Final votes are presented in Table 4.

**Table 4:** Consensus workshop outcomes.

Construct	Final vote (*n* = 12), *n* (%)						Consensus^a^
In the first 6 months after a stroke, what is the smallest indicator of important change on the anchor‐rating scale for [construct]?							
	Much worse	Slightly worse	No change	Slightly improved	Much improved	Completely recovered	
Language	0 (0%)	0 (0%)	1 (8%)	10 (83%)	1 (8%)	0 (0%)	Slightly improved
Communication	0 (0%)	0 (0%)	0 (0%)	11 (92%)	1 (8%)	0 (0%)	Slightly improved
Quality of life	0 (0%)	0 (0%)	1 (9%)	9 (75%)	2 (18%)	0 (0%)	Slightly improved
Emotional well‐being	0 (0%)	0 (0%)	0 (0%)	9 (75%)	3 (25%)	0 (0%)	Slightly improved
More than 6 months after a stroke, what is the smallest indicator of important change on the anchor‐rating scale for [construct]?							
	Much worse	Slightly worse	No change	Slightly improved	Much improved	Completely recovered	
Language	0 (0%)	0 (0%)	0 (0%)	5 (45%)	7 (55%)	0 (0%)	No consensus
Communication	0 (0%)	0 (0%)	0 (0%)	8 (67%)	4 (33%)	0 (0%)	No consensus
Emotional well‐being	0 (0%)	0 (0%)	0 (0%)	5 (45%)	7 (55%)	0 (0%)	No consensus
Quality of life	0 (0%)	0 (0%)	0 (0%)	4 (33%)	8 (67%)	0 (0%)	No consensus

^a^ Consensus is defined as ≥ 70% agreement, indicated in bold.

## 4 Discussion

This study involved people with aphasia and speech pathologists to explore how stakeholders conceptualise clinically meaningful changes in recovery after stroke. Whether a change is clinically meaningful is of key importance to evaluate the effectiveness of aphasia treatment and guide clinical decision‐making. People with aphasia are best placed to indicate whether a change feels important, so their inclusion in developing values of meaningful change is vital. The focus group findings provide an important foundation for the development of MIC values for core aphasia OMIs. Likewise, the quantitative findings have significant implications for interpreting treatment success. Consensus was reached that ‘slightly improved’ is an appropriate indicator of the smallest amount of meaningful change within the first 6 months after stroke. This agreement represents an initial threshold of clinically meaningful change on our anchor‐rating scales. Scores from OMIs are crucial to monitoring and improving services, and ultimately outcomes for people with aphasia [10, 35]. Therefore, establishing MIC values must be considered an important progression in aphasia outcome measurement.

Our study findings suggest that although the lived experience of aphasia is unique (Theme 1), there is commonality in the degree of change considered meaningful (Theme 2). The identified factors influencing perceptions of change (Theme 4) reinforce previous research findings that community participation [28], functional independence [36], autonomy [37], a sense of purpose and meaningful relationships [38] contribute to perceptions of ‘living well’ with aphasia. In addition, personal factors identified by speech pathologists, including emotional distress, impacts on participation and social factors, are known predictors of self‐rated quality of life in aphasia [39]. These findings suggest that there may be cohesion within the population of people with aphasia regarding the types of changes considered meaningful and these factors influencing why changes are perceived as important.

Speech pathologists identified the importance of collaboratively forming personally relevant goals (Theme 3). This finding is consistent with best practice clinical guidelines for aphasia management, which recommend collaborative goal setting [40]. The effectiveness of goal setting may contribute to whether patients perceive short‐term goals (i.e., small changes) as meaningful [37]. Dividing goals into achievable steps has been reported by speech pathologists as an important part of clinical practice and motivating patients aligning with the perspective of participants with aphasia, one of whom articulated ‘It's immensely important to step from one improvement to the next’ (PWA04) [41]. Notably, participants with aphasia emphasised the importance of speech pathologists celebrating the achievement of these short‐term goals. However, no known literature explores how clinicians celebrate goal attainment, which may form an important direction to enhance goal setting.

Delivery of person‐centred care relies on individualised information, such as the integration of outcome information, to be effective. Previous studies have sought to understand the important elements of person‐centred aphasia rehabilitation, including goal setting [42], shared decision‐making [43], therapy participation [44] and prognostication [42]. Collectively, this research suggests that by focusing on the relational aspects of care, clinicians can understand a patient holistically and then work in partnership towards a person's own goals [42, 43, 44]. Our findings affirm this approach and suggest that including measures of meaningful change in clinical practice may be a valuable addition to person‐centred care. In doing so, clinicians will be further supported to understand the needs of an individual and communicate outcomes in a contextualised way.

Baseline severity is a factor known to influence MIC values [45]. Because of this, we included questions about severity in the consensus process. However, participants with aphasia reported difficulty conceptualising characteristics to differentiate mild, moderate or severe aphasia so the questions were withdrawn. Further, project advisors with lived experience questioned the value of reporting the level of aphasia severity experienced by participants. This was raised as an unnecessary comparison between individuals as impairment severity may not equal the impact of aphasia on a person's usual activity. Together, these perspectives echo reported challenges of defining condition severity across qualitative research [46]. Further consideration and community consultation are required before exploring the predictive factor of aphasia severity on perceptions of clinically meaningful change.

Consensus was not reached on an appropriate indicator of the smallest amount of meaningful change beyond 6 months after stroke. Participants engaged in a collaborative discussion and deliberated over which response on the scale may be more reflective of their experiences. However, reducing these experiences to a single response on the anchor‐rating scale proved challenging. Participants expressed that the broad definition of ‘more than 6 months’ was more challenging to think about than the specific time frame of ‘less than 6 months’. These time frames were selected to reflect the timeline of the stroke recovery framework, in which the first 6 months after stroke are characterised as a period of rapid change and recovery, distinct from the longer chronic recovery period [47]. Nonetheless, voting patterns indicated participants overwhelmingly considered that either ‘slightly’ or ‘much’ improved was likely to be meaningful (Table 4). As with severity, more investigation is required to understand the perspectives of those with aphasia at different time points in recovery.

The challenges encountered by people with aphasia in rating their experiences reinforce the complexities and nuances of measuring within‐person change. The participants' difficulty calls attention to the limitations of patient‐rated scales, specifically that in isolation they are unlikely to provide all the information needed to make a judgement about clinically meaningful change. This observation is reflective of OMIs more broadly, as patient‐reported, performance‐based and clinician‐rated OMIs measure distinct information from each other. Although combining these data is valuable, no single measure captures sufficient information to understand the impact of aphasia on a person [13, 35]. Accordingly, clinicians should exercise caution in using any single OMI to measure aphasia outcomes. Integrating information from multiple sources is likely to enrich conversations with patients when forming goals, selecting treatments and monitoring progress [13, 48].

Altogether, the combination of qualitative and quantitative findings underscores the necessity of a range of values that represent meaningful changes in stroke recovery. Across health conditions, patient demographics, baseline severity, treatment type and follow‐up period can all influence the estimation of MIC values [49, 50]. As such, MIC values are not considered to be a universally accepted score. Rather, they require clinicians to evaluate the population and methodology used to establish each MIC value for an OMI to ensure its relevance to the individual patient.

### 4.1 Strengths and Limitations

This exploratory study enhances knowledge in aphasia outcome measurement by providing insights from clinicians and people with lived experience of clinically meaningful changes. A mixed‐methods approach was undertaken, which provides preliminary evidence towards the development of MIC values for core aphasia OMIs. This evidence was further enhanced by establishing a stakeholder‐nominated threshold of minimal importance in the first 6 months after stroke on an anchor‐rating scale for four core constructs.

The study participant sample represents a range of people with aphasia and clinicians working in aphasia rehabilitation. Given that a primary finding was that meaningful change is different for every person, it remains unclear whether the experiences and views expressed by the study's participants are representative of the broader population of stakeholders, or whether these findings would replicate in a larger, more diverse, sample. The anchor‐rating scale was developed based on existing research recommendations. Although we did seek consumer feedback, the scale may not directly reflect how participants with aphasia perceived their recovery. Validation of the scale with a larger sample of people with aphasia is a potential future step towards establishing MIC values of core aphasia OMIs.

Our results may be biased towards the perspectives of English‐speaking patients within the subacute and chronic stages of stroke recovery. Future research should consider recruitment methods that represent a broader range of geographically and culturally diverse experiences, including First Nations peoples and people living in rural and remote areas. Most participants with aphasia indicated that they had experienced multiple conditions as a result of their stroke (Table 2). Depression, anxiety and changes to cognitive functions are common after stroke and may potentially impact a person's perception and insight into their recovery [51]. The potential impact of these factors on participants' perceptions of change was not explored in this study.

Clinically meaningful changes in this study were explored in the context of improvements across the key constructs. However, previous research indicates what is considered meaningful may differ depending on the direction of change during recovery [45]. Therefore, differences in clinically meaningful effects between improvement and worsening warrant future exploration.

### 4.2 Future Directions

Based on the findings from this research, the indicator of ‘slightly improved’ will be applied to patient‐reported responses on the anchor‐rating scale in a planned study [52]. Study participants will complete the four OMIs of the ROMA COS at baseline, and again after usual care aphasia treatments in rehabilitation settings. At the post‐treatment time point, participants will be categorised as ‘importantly improved’ if they rated their changes as slightly improved or higher on the anchor‐rating scale. Scores lower than ‘slightly improved’ will be categorised as ‘not importantly improved’. Change scores from the ROMA COS instruments will be analysed within these groups, following the anchor‐based, predictive modelling approach to establishing MIC values established by Terluin et al. [53]. In this way, objective values of clinically meaningful change may be established for core aphasia OMIs, and any differences in meaningful change between individual patients or patient subgroups (e.g., differences mediated by personal or treatment‐related factors) may be identified.

## 5 Conclusion

A mixed‐methods investigation was conducted to examine the clinical meaningfulness of changes in aphasia recovery. Four themes were identified, which characterise clinically meaningful changes in aphasia recovery as follows: (1) different for every single person, (2) small but continuous improvements, (3) measured by progress towards personal goals and (4) influenced by personal factors. The perspectives shared affirm that a person‐centred approach to rehabilitation, goal setting in particular, supports people with aphasia to achieve meaningful changes during their recovery. Participants found nominating a response on a scale that adequately described their experiences of clinically meaningful change to be a challenging process. Nonetheless, the findings indicate that integrating patient perceptions of change with OMI scores may be a valuable addition to patient‐centred aphasia rehabilitation practices.

## Author Contributions

**Sally Zingelman:** conceptualisation, writing–original draft, methodology, validation, visualisation, writing–review and editing, formal analysis, project administration, resources, data curation, investigation. **Dominique A. Cadilhac:** conceptualisation, writing–review and editing, supervision, methodology, formal analysis. **Joosup Kim:** conceptualisation, writing–review and editing, supervision, methodology, formal analysis. **Marissa Stone:** validation, writing–review and editing, methodology. **Sam Harvey:** writing–review and editing, supervision, methodology. **Carolyn Unsworth:** methodology, formal analysis, writing–review and editing, investigation. **Robyn O'Halloran:** methodology, writing–review and editing, formal analysis. **Deborah Hersh:** methodology, writing–review and editing, formal analysis. **Kathryn Mainstone:** conceptualisation, writing–review and editing, formal analysis, visualisation. **Sarah J. Wallace:** conceptualisation, investigation, funding acquisition, methodology, writing–review and editing, formal analysis, supervision, visualisation, project administration.

## Ethics Statement

Ethical approval was obtained from the University of Queensland's Human Research Ethics Committee (2022HE001946).

## Consent

Participants were recruited through stroke and aphasia professional organisations, including the Australian Aphasia Association, Queensland Aphasia Research Centre, Stroke Foundation, the Centre of Research Excellence in Aphasia Recovery and Rehabilitation and the Speech Pathology Email ChatS (SPECS) Google group. Written project information was provided and presented using accessible language and formatting for people living with aphasia [42]. Interested individuals were invited to submit an expression of interest via Qualtrics. Prospective participants with aphasia then attended an individual videoconferencing meeting with the first researcher. In this meeting, the participant information and consent were discussed verbally with pictorial supports, with opportunities to ask questions before obtaining informed consent. Prospective speech pathology participants were provided with written consent forms via email.

## Conflicts of Interest

The authors declare no conflicts of interest.

## Supporting information

Supporting information.

Supporting information.

## Data Availability

Data that support the findings of this study are not publicly available. Anonymised data may be made available from the corresponding author upon reasonable request.
