# Supplementary material for: ‘A Meaningful Difference, but Not Ultimately the Difference I Would Want’: A Mixed‐Methods Approach to Explore and Benchmark Clinically Meaningful Changes in Aphasia Recovery
Source: Health Expect. 2024 Aug 6;27(4):e14169. doi: 10.1111/hex.14169 (PMC11302794; doi:10.1111/hex.14169)
Supplement: Supplementary file 1 — Supporting information. [file HEX-27-e14169-s001.docx]

| **Supplementary File 1: Study Patient and Public Contributions** | |
| --- | --- |
| **Study Phase and descriptions*** | **Consumer activity within the study** |
| *Stage 1: Foundation*   - Collaboration. - Recruitment. - Informed consent. - Patient/partner training - Communication links | - The Australian Aphasia Association (national organisation) and the Queensland Aphasia Research Centre are research partners on this project. - Consent to participate as a consumer investigator or advisor was obtained directly from the consumer. - Meetings between members of the research team and the consumer advisory committee were held to:   - Establish rapport within the group.   - Discuss financial compensation.   - Collaboratively develop terms of reference, roles, and responsibilities.   - Set an accessible agenda, including breaks.   - Define how consumers would be actively involved. - Information about the research process was presented to consumer advisors in an accessible format. Opportunities for questions was provided. - Communication between the research team, consumer investigators and consumer advisors adhere to aphasia friendly formatting principles, including email content, agendas, and presentation materials. |
| *Phase 2: Development*   - Conceptualisation. - Research priorities. - Co-design methodology. - Proposal development. | - Generated research ideas from conversations with research investigators. - Stated whether the answers to research questions were important to the views and opinions of people with aphasia. - Ensured the purpose of the study was easy for all partners to understand. - Ensured the research proposal stated how people with aphasia would be involved in the research. - Named as research partners. |

| **Supplementary File 1 Cont…** | |
| --- | --- |
| **Study Phase and descriptions*** | **Consumer activity within the study** |
| *Phase 3: Translational*   - Outcomes - Interpretation - Dissemination - Sustainability | - Critically appraised patient-facing materials for study participants. - Assisted in conducting consensus workshop. - Involved in interpreting qualitative data. Preliminary themes were presented, and the grouping and labelling of data was reviewed collaboratively. - Co- designed aphasia-friendly handout of results (Supplementary File 1). - Conceptualised a dissemination strategy to the community of people with aphasia. - Critically reviewed conference abstracts and presentations. - Future plans to co-present results. |

*Note*: *Descriptions summarised from the PAOLI framework.^40^*
