# Supplementary material for: ‘A Meaningful Difference, but Not Ultimately the Difference I Would Want’: A Mixed‐Methods Approach to Explore and Benchmark Clinically Meaningful Changes in Aphasia Recovery
Source: Health Expect. 2024 Aug 6;27(4):e14169. doi: 10.1111/hex.14169 (PMC11302794; doi:10.1111/hex.14169)
Supplement: Supplementary file 2 — Supporting information. [file HEX-27-e14169-s002.pdf]

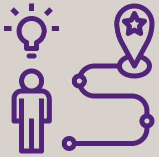

What you can do

You can set goals for recovery:

- **Set goals together** with your speech pathologist.
- **Personal goals** may help you **make meaningful changes**.

Loved ones and colleagues can help by:

- **Keeping track** of the **changes** you notice.
- Talking about the **impact aphasia has** on **participating** in everyday life.
- **Celebrating progress**!

The research team gratefully acknowledges funding support received from Medical Research Futures Fund MRFF 2021 Cardiovascular Health Grant Opportunity: The Right Treatment for the Right Person at the Right Time. Driving High-Value Aphasia Care through Meaningful Health System Monitoring (MRF2016134) and the Australian Government via Research Training Program scholarships.

Aphasia information

Queensland Aphasia Research Centre
https://shrs.uq.edu.au/qarc
qarc@uq.edu.au

Australian Aphasia Association
aphasia.org.au
questions@aphasia.org.au

Stroke Foundation
strokefoundation.org.au
strokeline@strokefoundation.org.au

Enable Me online stroke community
https://enableme.org.au/

Australian Aphasia Rehabilitation Pathway
Information about setting goals.
www.aphasiapathway.com.au

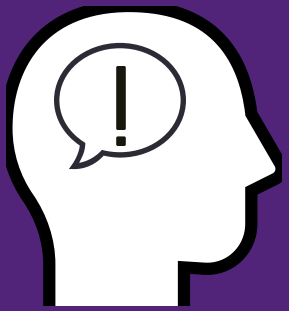

Understanding important changes in aphasia recovery

A research summary
S. Zingelman, D.A Cadilhac, J. Kim, M. Stone, S.Harvey, C. Unsworth, R. O'Halloran, D. Hersh, K Mainstone, and S. J. Wallace (2024).
together with
B. Lewis, P.Mainstone, M.Stewart, and M. Yutzuss.

The University Of Queensland
Australia
Create Change

Queensland Aphasia Research Centre

The question

- People with aphasia complete **tests before therapy** and **again after therapy**.
- Test scores **give information** about a **person's abilities** and **feelings**.
- Test **scores** can **change** with **time and therapy**.

We wanted to know: **what are important changes in aphasia recovery?**

- In the **future** we will use this information to make **test scores easier to understand.**

What we did.

- We met with **people with aphasia** and **speech pathologists.**
- We **asked** their **opinions**.

What we found

People told us that:

Small continuous improvements are important.

- **Celebrating small improvements** can be **motivating** in rehabilitation.
- **Short-term goals** are **meaningful steps** to reaching a long-term goal.

"It's immensely important to step from one improvement to the next."

'Neil' (56 years old, 8 months post-stroke)

Important changes

Important changes are:

Different for every single person.

- **Everyone with aphasia is different.**
- Changes in **language, communication,** and **quality of life** can **feel important**.
- The **type of change** that is feels important is **unique to you**.
- **Adjusting** to **'the new normal'** after stroke can feel important.
- It's **important to talk** to your speech pathologist **about the impact of aphasia** on your **usual activities**.
